# Venetoclax (ABT-199) Might Act as a Perpetrator in Pharmacokinetic Drug–Drug Interactions

**DOI:** 10.3390/pharmaceutics8010005

**Published:** 2016-02-24

**Authors:** Johanna Weiss, Thomas Gajek, Bruno Christian Köhler, Walter Emil Haefeli

**Affiliations:** 1 Department of Clinical Pharmacology and Pharmacoepidemiology, University of Heidelberg, Im Neuenheimer Feld 410, 69120 Heidelberg, Germany; gajek.thomas@googlemail.com (T.G.); walter.emil.haefeli@med.uni-heidelberg.de (W.E.H.); 2 National Centre for Tumour Diseases, Department of Medical Oncology, Internal Medicine VI, University of Heidelberg, Im Neuenheimer Feld 460, 69120 Heidelberg, Germany; bruno.koehler@nct-heidelberg.de

**Keywords:** ABT-199, venetoclax, CYPs, drug transporters, drug–drug interaction

## Abstract

Venetoclax (ABT-199) represents a specific B-cell lymphoma 2 (Bcl-2) inhibitor that is currently under development for the treatment of lymphoid malignancies. So far, there is no published information on its interaction potential with important drug metabolizing enzymes and drug transporters, or its efficacy in multidrug resistant (MDR) cells. We therefore scrutinized its drug–drug interaction potential *in vitro*. Inhibition of cytochrome P450 enzymes (CYPs) was quantified by commercial kits. Inhibition of drug transporters (P-glycoprotein (P-gp, *ABCB1*), breast cancer resistance protein (BCRP), and organic anion transporting polypeptides (OATPs)) was evaluated by the use of fluorescent probe substrates. Induction of drug transporters and drug metabolizing enzymes was quantified by real-time RT-PCR. The efficacy of venetoclax in MDR cells lines was evaluated with proliferation assays. Venetoclax moderately inhibited P-gp, BCRP, OATP1B1, OATP1B3, CYP3A4, and CYP2C19, whereas CYP2B6 activity was increased. Venetoclax induced the mRNA expression of *CYP1A1*, *CYP1A2*, *UGT1A3*, and *UGT1A9*. In contrast, expression of *ABCB1* was suppressed, which might revert tumor resistance towards antineoplastic P-gp substrates. P-gp over-expression led to reduced antiproliferative effects of venetoclax. Effective concentrations for inhibition and induction lay in the range of maximum plasma concentrations of venetoclax, indicating that it might act as a perpetrator drug in pharmacokinetic drug–drug interactions.

## 1. Introduction

Venetoclax (ABT-199) is a specific oral B-cell lymphoma 2 (Bcl-2) inhibitor currently in clinical trials for the treatment of chronic lymphatic leukemia (CLL), acute myelogenous leukemia, small lymphocytic lymphomas, and multiple myeloma [1,2,3]. It recently received a breakthrough therapy designation by the Food and Drug Administration (FDA) for the therapy of pre-treated CLL with 17p deletion [2]. Bcl-2 represents an antiapoptotic protein playing an important role in tumorigenesis and chemoresistance, which is often over-expressed in hematopoietic malignancies [1,2]. In contrast to navitoclax (ABT-263), venetoclax does not inhibit Bcl-x_L_, thus harboring no risk for thrombocytopenia [3,4].

Safety and effectiveness of antineoplastic drugs can be critically altered by drug-drug interactions. Increased or reduced activity of drug metabolizing enzymes and drug transporters can lead to decreased or increased exposure provoking reduced efficacy or toxic side effects. So far, for venetoclax, there are no published data at all concerning its interaction with drug metabolizing enzymes and drug transporters. Moreover, it is unknown whether over-expression of multidrug resistance (MDR) proteins like P-glycoprotein (P-gp, *ABCB1*) provokes resistance towards this antineoplastic drug.

We therefore underwent *in vitro* examinations to determine whether (1) venetoclax inhibits important drug transporters modulating intestinal absorption or hepatic uptake (P-gp, breast cancer resistance protein (BCRP/*ABCG2*), organic anion transporting polypeptides 1B1 and 1B3 (OATP1B1, OATP1B3)), (2) it inhibits important CYPs (CYP1A2, CYP2B6, CYP2C19, CYP2D6, and CYP3A4), (3) it influences the expression of relevant drug metabolizing enzymes and drug transporters, and (4) it retains its efficacy in MDR cell lines.

## 2. Materials and Methods

### 2.1. Materials

Culture media, medium supplements, buffers, the GenElute™ Mammalian Total RNA Miniprep Kit, fumitremorgin C (FTC), and the anti-β-actin antibody (Clone AC-74) were purchased from Sigma–Aldrich (Taufkirchen, Germany). Fetal calf serum (FCS) was purchased from Biochrom (Berlin, Germany). Crystal violet, dimethyl sulfoxide (DMSO), TRIS (2-amino-2-(hydroxymethyl)-propan-1,3-diol), sodium dodecyl sulfate (SDS), glycerol, Tween^®^20, dithiothreitol (DTT), rifampicin, sodium dodecyl sulfate (SDS), and Triton^®^ X-100 were purchased from AppliChem (Darmstadt, Germany). Bromphenol blue and PCR microplates were purchased from Biomol (Hamburg, Germany). Calcein acetoxymethylester (calcein-AM) was obtained from Invitrogen (Karlsruhe, Germany), pheophorbide A from Frontier Scientific Europe (Carnforth, UK), and 8-fluorescein-cAMP (8-FcA) from BIOLOG Life Science Institute (Bremen, Germany). Pheophorbide A (PhA) was obtained from Frontier Scientific Europe (Carnforth, UK). Nitrocellulose membranes (Optitran BA-S 85) were obtained from Schleicher & Schuell BioScience (Dassel, Germany). The antibody against human P-gp clone C219 was obtained from Calbiochem (Darmstadt, Germany), and the antibody against CYP1A2 (clone D-3) and the corresponding secondary donkey anti-goat antibody were from Santa Cruz (Heidelberg, Germany). The secondary anti-mouse antibody was obtained from GE Healthcare (Freiburg, Germany). Slim-Fast^®^ was obtained from Allpharm Vertriebs GmbH (Messel, Germany). The SuperSignal^®^West Pico Chemiluminescent Substrate Kit was from Pierce (Rockford, IL, USA). Cell-culturing bottles were supplied by Greiner (Frickenhausen, Germany) and 96-well microtiter plates by Nunc (Wiesbaden, Germany). White 96-well plates with clear bottom and clear lid for luminometry were supplied by Greiner (Frickenhausen, Germany). The RevertAid™ H Minus First Strand cDNA Synthesis Kit was obtained from Fermentas (St. Leon-Rot, Germany). Absolute QPCR SYBR Green Mix was obtained from ABgene (Hamburg, Germany) and the QuantiTect^®^ Primer Assay for UGT1A3 from Qiagen (Hilden, Germany). Primers were synthesized by Eurofins MWG Operon (Ebersberg, Germany). The Steady-Glo™ Luciferase Assay and the P450-Glo CYP1A2 Screening System were obtained from Promega Corporation (Madison, WI, USA). The CYP2D6/AMMC, the CYP2B6/EFC, the CYP2C19/CEC, and the CYP3A4/BFC High Throughput Inhibitor Screening Kits were purchased from Becton Dickinson Biosciences (Heidelberg, Germany). Zosuquidar (LY335979) was obtained from Eli Lilly (Bad Homburg, Germany). Venetoclax was purchased from Sequoia Research Products (Pangbourne, UK).

### 2.2. Cell Lines

**MDCKII cells.** The transporter over-expressing cell lines MDCKII-MDR1 [5], MDCKII-MRP2 [6], and MDCKII-BCRP [7] and the corresponding parental cell line MDCKII were used to assess the sensitivity of cells with and without P-gp, multidrug resistance-associated protein 2 (MRP2/ABCC2), or BCRP expression. Moreover, MDCKII-BCRP cells were used to assess BCRP inhibition by venetoclax. MDCKII over-expressing cell lines were kindly provided by A. H. Schinkel and P. Borst (Amsterdam, The Netherlands). The parental cell line MDCKII (available at ATCC, Manassas, VA, USA) was used as a control. The cells were cultured in Dulbecco’s Modified Eagle’s Medium (DMEM) containing 10% FCS, 2 mM of glutamine, 100 U/mL of penicillin, and 100 µg/mL of streptomycin sulfate.

**LLC-PK1 and L-MDR1 cells.** L-MDR1 cells, a porcine kidney epithelial cell line over-expressing the human *ABCB1* gene [8] and the corresponding parental control cell line LLC-PK1 (available from ATCC, Manassas, VA, USA) were used to assess the sensitivity of cells with and without P-gp over-expression. L-MDR1 cells were kindly provided by A. H. Schinkel (The Netherlands Cancer Institute, Division of Experimental Therapy, Amsterdam, The Netherlands). The cells were cultured under standard cell culture conditions with medium M199 supplemented with 10% FCS, 2 mM of glutamine, 100 U/mL of penicillin, and 100 µg/mL of streptomycin sulfate. To maintain P-gp/*ABCB1* expression the culture medium for L-MDR1 was supplemented with 0.64 µM of vincristine. One day before seeding the cells for the growth inhibition assay, both cell lines were fed with a vincristine-free culture medium.

**P388 and P388/dx cells.** For testing P-gp inhibition, the murine monocytic leukemia cell line P388/dx over-expressing murine mdr1a/b and the corresponding parental cell line P388 were used [9]. Both cell lines were kindly provided by D. Ballinari (Pharmacia & Upjohn, Milano, Italy). The cells were cultured under standard cell culture conditions with an RPMI 1640 medium supplemented with 10% FCS, 2 mM of glutamine, 500 mM of β-mercaptoethanol, 100 U/mL of penicillin, and 100 µg/mL of streptomycin sulfate. For maintaining P-gp expression, the culture medium for P388/dx was supplemented with 0.43 µM of doxorubicin. One day before the assay, both cell lines were fed with a doxorubicin-free culture medium.

**HEK293 cells.** For assessing inhibition of OATP1B1 and OATP1B3, the human embryonic kidney cell line HEK293 stably transfected with OATP1B1 (HEK-OATP1B1), OATP1B3 (HEK-OATP1B3), or the empty control vector (HEK293-VCG418) were used [10,11]. Cells were cultured under standard cell culture conditions with DMEM supplemented with 10% FCS, 2 mM of glutamine, 100 U/mL of penicillin, 100 µg/mL of streptomycin sulfate, and 800 µg/mL of G418 to maintain over-expression. Cells were kindly provided by D. Keppler (German Cancer Research Centre, Heidelberg, Germany).

**LS180 cells.** The human colon adenocarcinoma cell line LS180 (available at ATCC, Manassas, VA, USA) is one of the standard models for investigating pregnane X receptor (PXR) and aryl hydrocarbon receptor (AhR) mediated induction [12,13,14,15,16,17,18,19] and was thus used for induction experiments. Cells were cultured under standard cell culture conditions with DMEM supplemented with 10% FCS, 2 mM of glutamine, 100 U/mL of penicillin, 100 µg/mL of streptomycin sulfate, and 0.1 mM of nonessential amino acids.

**AZ-AHR cells**. The human hepatoma cell line HepG2 stably transfected with a construct containing several AhR binding sites upstream of luciferase reporter gene [20] was used to investigate whether venetoclax can activate AhR. Cells were kindly provided by Z. Dvorak (Palacky University, Olomouc, Czech Republic).

### 2.3. Cytotoxicity Assay

Prior to P-gp, BCRP, and OATP inhibition assays, venetoclax was tested in the respective cell lines for cytotoxic effects with the Cytotoxicity Detection Kit (Roche Applied Science, Mannheim, Germany). Venetoclax was not cytotoxic up to 100 µM in all cell lines used.

### 2.4. P-gp Inhibition Assay

P-gp inhibition was assessed with the calcein assay in P388/dx cells as described previously [21]. Inhibition of P-gp increases intracellular calcein fluorescence in these cells. Each venetoclax concentration (0.005–50 µM) was tested in octuplet, and the experiment was performed in quadruplicate.

### 2.5. BCRP Inhibition Assay

Flow cytometric BCRP inhibition assays were conducted in control (MDCKII) and BCRP over-expressing cells (MDCKII-BCRP) using PhA as a fluorescent BCRP substrate as described and validated previously [22]. Inhibition of BCRP leads to an increase in intracellular PhA concentrations. Each experiment was performed in triplicate. Venetoclax was tested from 0.1 up to 100 µM.

### 2.6. OATP Inhibition Assay

Inhibition of OATP1B1 and OATP1B3 was analyzed in HEK-OATP1B1 and HEK-OATP1B3 cells by quantifying the uptake of the fluorescent substrate 8-FcA by flow cytometry as described previously [12]. HEK293-VCG418 cells were used as a control. Inhibition of OATPs leads to a reduced uptake of 8-FcA into the cells. Each experiment was performed at least in triplicate, and venetoclax was tested from 0.05 up to 100 µM.

### 2.7. Inhibition of CYPs

Inhibition studies for CYP2B6, CYP2D6, CYP2C19 and CYP3A4 were performed with the CYP2B6/EFC, CYP2D6/AMMC, the CYP2C19/CEC, and the CYP3A4/BFC High Throughput Inhibitor Screening Kit according to the manufacturer’s instructions. The kits contain the respective recombinant CYP and fluorogenic substrates, which are blocked dyes emitting a fluorescence signal when cleaved by the enzyme. For testing CYP1A2 inhibition, the P450-Glo CYP1A2 Screening System was used according to the manufacturer’s instructions. The kit contains the luminogenic CYP1A2 substrate luciferin-ME, which is converted by CYP1A2 into luciferin-generating light when incubated with the luciferin detection reagent of the kit. Eight concentrations of venetoclax in duplicates (0.009–20 µM) were tested in the fluorescence assays, and eight concentrations in triplicate (0.05–100 µM) were tested for the luminogenic assay. Each experiment was conducted in triplicate.

### 2.8. Growth Inhibition (Proliferation) Assay

Growth inhibition assays in LS180 cells were conducted to determine suitable maximum concentrations for the induction assay without profound antiproliferative effects. Proliferation was quantified by crystal violet staining of the surviving cells as described previously [23]. Each experiment was performed at least in triplicate with *n* = 8 wells for each concentration (0.1–100 µM). The IC_20_ for proliferation inhibition by venetoclax was 8.4 ± 1.0 µM; thus, the maximum concentration for the induction assay was set to 10 µM, ensuring that about 80% of the cells will survive.

Growth inhibition assays in MDCKII, and their MRP2 or BCRP over-expressing counterparts (MDCKII-MDR1, MDCKII-BCRP) and in LLC-PK1 and the corresponding P-gp over-expressing cell line L-MDR1 were conducted to evaluate whether venetoclax sustains its efficacy in MDR cell lines. To confirm the observed involvement of P-gp in the resistance of L-MDR1 cells towards venetoclax, the specific P-gp inhibitor LY335979 (1 µM) was used.

### 2.9. Induction Assay

For the induction experiments, LS180 cells were seeded in 75 cm^2^ culturing flasks and incubated for three days. Cells were then treated with a culture medium containing venetoclax (0.01–10 µM) in quintuplicate for four consecutive days. Rifampicin (20 µM) served as a positive control for PXR-driven genes and culture medium as a negative control. All incubation solutions were adjusted to 0.02% DMSO. After harvesting, cells were split for RNA and protein extraction.

### 2.10. Quantification of mRNA Expression by Real-Time RT-PCR

RNA was isolated using the GenElute™ Mammalian Total RNA Miniprep Kit, and cDNA was synthesized with the RevertAid™ H Minus First Strand cDNA Synthesis Kit according to the manufacturer's instructions. mRNA expression was quantified by real-time RT-PCR with the LightCycler^®^ 480 (Roche Applied Science, Mannheim, Germany) as described previously [15,24]. Primer sequences were also published previously [15,25,26,27]. The most suitable housekeeping gene for normalization in LS180 cells was identified using geNorm (version 3.4, Center for Medical Genetics, Ghent, Belgium), which determines the most stable reference gene from a set of tested genes in a given cDNA sample panel [28]. Among a panel of 7 housekeeping genes tested, *β2-microglobulin* (*β2mg*) proved to be the most stable gene in LS180 cells under the selected experimental conditions. Data were evaluated via calibrator-normalized relative quantification with efficiency correction using the LightCycler^®^ 480 software version 1.5 (Roche Applied Science, Mannheim, Germany). All samples were amplified in duplicate. The following target genes were quantified: *CYP1A1*, *CYP1A2*, *CYP3A4*, *ABCB1*, *ABCC2*, *ABCG2*, *SLCO1B1* (coding for OATP1B1), *UDP-glucuronosyltransferase 1A3 (UGT1A3)*, and *UGT1A9*.

### 2.11. Western Blot Analysis of P-gp and CYP1A2 Protein Expression

To exemplarily verify the effects of venetoclax on mRNA expression, protein expressions of P-gp and CYP1A2 were analyzed in triplicate by SDS polyacrylamide gel electrophoresis (SDS-PAGE) and Western blotting. In brief, cell lysates containing 20 µg protein were mixed with 5× sample buffer (containing Tris–HCl, SDS, DTT, bromophenol blue, and glycerol) and subjected to 10% SDS-PAGE. Afterwards, proteins were electrotransferred to nitrocellulose nitrate membranes. Blots were blocked by incubation for 20–40 min with 5% Slim-Fast^®^ (*w*/*v*) in phosphate-buffered saline containing 0.1% Tween^®^20. Immunoblot analysis was carried out with antibodies raised against human P-gp (diluted 1:100 in Tris-buffered saline containing 0.1% Tween^®^20 (TBST)), human CYP1A2 (clone D-3, diluted 1:200), or β-actin (Clone AC-74; diluted 1:40,000). After extensive washing of the membranes, blots were incubated with horseradish peroxidase-linked secondary antibodies. Bands were visualized by enhanced chemiluminescence using the SuperSignal^®^West Pico Chemiluminescent Substrate Kit and recorded by FluorChem Q SA AlphaView Version 3.2.2, Cell Biosciences (Santa Clara, CA, USA).

### 2.12. AhR Reporter Gene Assay

To test whether the induction of CYP1A1 or UGTs can be attributed to AhR activation by venetoclax, an AhR reporter gene assay was applied. 60,000 AZ-AhR cells were seeded into each well of 96-well plates with clear bottom and clear lid for luminometry. After incubation for 24 h, cells were treated in triplicate with venetoclax (0.1–10 µM) or vehicle control (0.2% DMSO) for a further 24 h. The assay was performed with the Steady-Glo™ Luciferase Assay System according to the manufacturer’s instructions. Drug-induced increases of AhR receptor activity were normalized to activity of non-drug treated controls, which was set to 1 (= 100%). The experiment was performed in triplicate.

### 2.13. Statistical Analysis

Data were analyzed using GraphPad Prism Version 6.02 and InStat Version 3.06 (GraphPad Software, San Diego, CA, USA). The differences in mRNA expression following incubation with test compounds and vehicle controls and the differences in antiproliferative effects were tested using ANOVA with Dunnett’s *post hoc* test. *p* ≤ 0.05 was considered significant.

## 3. Results

### 3.1. Venetoclax Inhibits P-gp

Venetoclax increased intracellular calcein fluorescence concentration-dependently in P-gp over-expressing P388/dx cells, but not in the parental cell line P388, indicating P-gp inhibition (Table 1).

### 3.2. Venetoclax Inhibits BCRP

Venetoclax concentration-dependently increased intracellular PhA concentration in MDCKII cells over-expressing BCRP (MDCKII-BCRP), but not in the parental cell line MDCKII, indicating BCRP inhibition (Table 1).

### 3.3. Venetoclax Inhibits OATP1B1 and OATP1B3

Venetoclax concentration-dependently inhibited 8-FcA uptake in HEK-OATP1B1 and HEK-OAPT1B3 cells, but not in the control cell line HEK293-VCG418, clearly indicating inhibition of OATP1B1 and OATP1B3 (Table 1).

### 3.4. Inhibition of CYPs by Venetoclax

**Figure 1:**
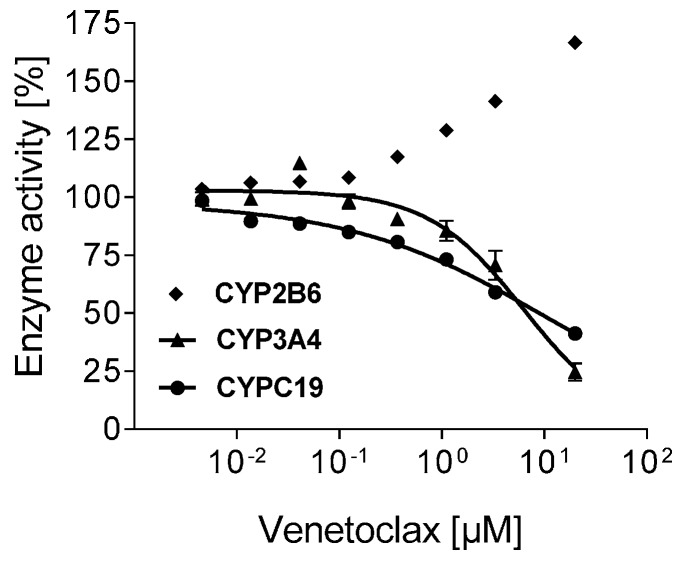
Inhibition of CYPs. Concentration-dependent effect of venetoclax (0.01–20 µM) on the enzyme activity of CYP2B6, CYP2C19, and CYP3A4. Data are expressed as mean ± SEM for *n* = 3 biological replicates measured in duplicate. CYPs with no inhibition (CYP1A2, CYP2D6) are not depicted.

Venetoclax inhibited CYP2C19 and CYP3A4 in the lower micromolar range (Table 1), whereas CYP2D6 and CYP1A2 were unchanged and activity of CYP2B6 was increased (1.7-fold at 20 µM) (Figure 1).

### 3.5. Influence of Venetoclax on the Expression of Drug Metabolising Enzymes and Drug Transporters

**Figure 2:**
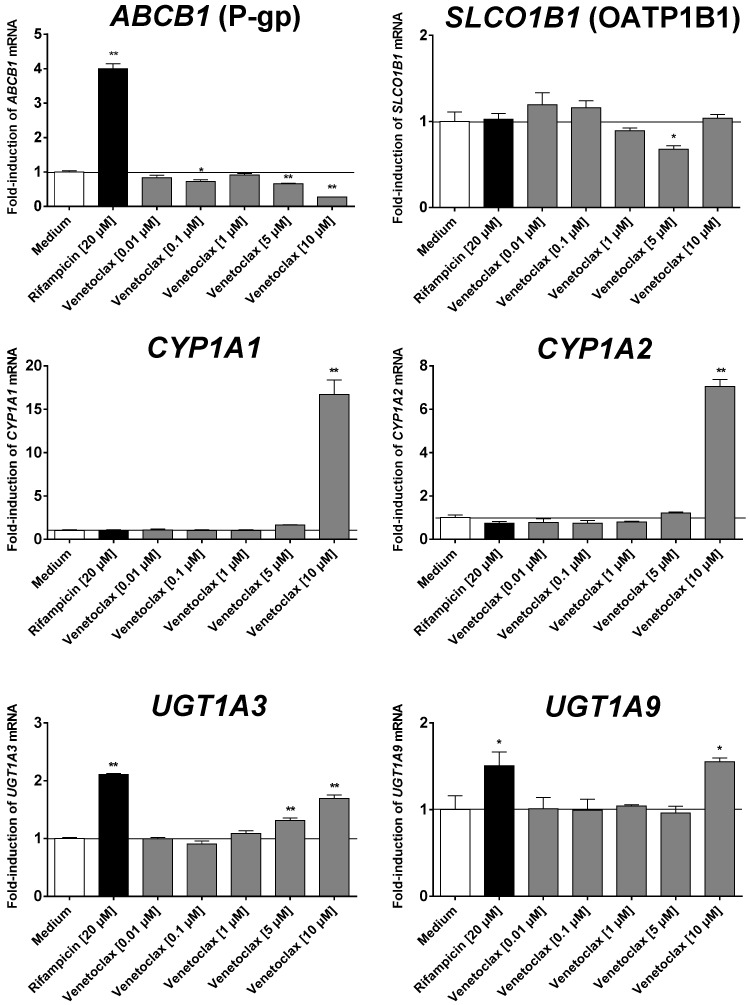
Induction assay. Concentration-dependent effect of venetoclax (0.01–10 µM) and 20 µM of rifampicin (positive control for PXR-driven genes) after four days on mRNA expression in LS180 cells compared to untreated medium control. Expression data were normalized to the housekeeping gene *β2mg*. Data are expressed as mean ± SEM for *n* = 5 biological replicates and normalized to the medium control (set to 1). Differences in mRNA expression to the medium control were tested using ANOVA with Dunnett’s *post hoc* test. * *p* < 0.05, ** *p* < 0.01.

At the highest concentration tested (10 µM), venetoclax induced mRNA expression of *CYP1A1*, *CYP1A2*, *UGT1A3*, and *UGT1A9* (Figure 2). In contrast, mRNA expression of *ABCB1* was suppressed at higher concentrations as was *SLCO1B1* expression at 5 µM of venetoclax. mRNA expressions of *CYP3A4*, *ABCG2*, and *ABCC2* were unchanged (data not shown).

**Figure 3:**
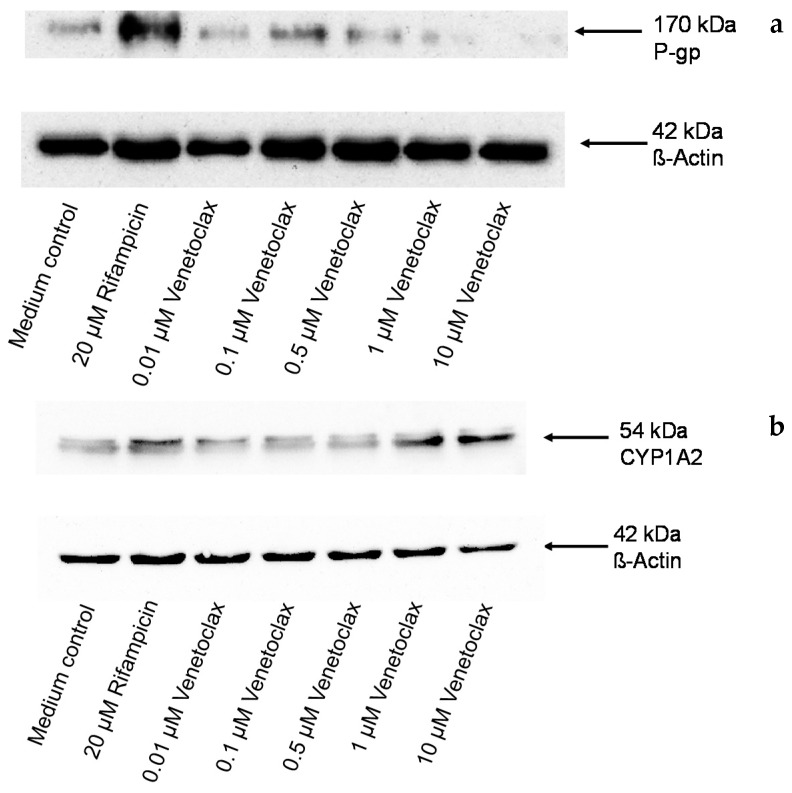
Western blot. Concentration-dependent effect of venetoclax (0.01–10 µM) and 20 µM of rifampicin (positive control for P-gp) after four days on P-gp (**a**) and CYP1A2 (**b**) protein expression in LS180 cells compared to untreated medium control. β-actin served as a loading control. Depicted is one blot of a series of three.

Suppression of P-gp expression at higher venetoclax concentrations was verified at the protein level. The Western blot analysis clearly demonstrated suppression of P-gp protein expression at 1 and 10 µM (Figure 3a). The Western blot analysis also demonstrated that induction of CYP1A2 by venetoclax translates into protein expression (Figure 3b).

### 3.6. Venetoclax Does not Activate AhR

Venetoclax did not increase luciferase activity in the AhR reporter gene assay up to 10 µM, indicating that it does not influence AhR activity.

### 3.7. Efficacy in MDR Cell Lines

**Figure 4:**
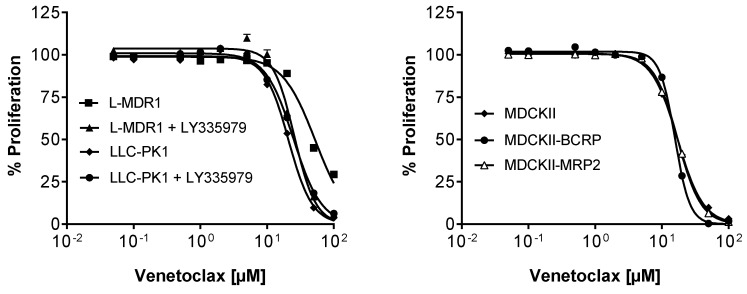
Growth inhibition assay. Concentration-dependent effect of venetoclax (0.05–100 µM) on the proliferation of the P-gp over-expressing cell line L-MDR1, the corresponding parental cell line LLC-PK1 with and without the specific P-gp inhibitor LY335979 (left) and of the BCRP or MPR2 over-expressing cell lines (MDCKII-BCRP, MDCKII-MRP2) and the corresponding parental cell line MDCKII (right). Each curve depicts the results of four experiments with each concentration tested in octuplet. Data are expressed as mean ± SEM for *n* = 32 wells.

Growth inhibition by venetoclax did not differ in BCRP (MDCKII-BCRP) or MPR2 (MDCKII-MRP2) over-expressing cell lines, suggesting that it retains its efficacy in tumors with BCRP or MRP2 over-expression (MDCKII, IC_50_ = 15.2 ± 2.4 µM; MDCKII-BCRP, IC_50_ = 15.7 ± 0.4 µM; MDCKII-MRP2, IC_50_ = 17.2 ± 1.8 µM) (Figure 4). In contrast, P-gp over-expressing L-MDR1 cells were more resistant to venetoclax than the control cell line LLC-PK1 (*p* < 0.01) and the resistance could be abolished in presence of the P-gp specific inhibitor LY335979 (LLC-PK1, IC_50_ = 21.0 ± 0.7 µM; LLC-PK1 + LY335979, IC_50_ = 24.9 ± 1.0 µM; L-MDR1, IC_50_ = 53.1 ± 4.1 µM; L-MDR1 + LY335979, IC_50_ = 25.7 ± 4.1 µM) (Figure 4). This indicates that P-gp over-expression can diminish the efficacy of venetoclax.

## 4. Discussion

Inhibition of Bcl-2 represents a promising new concept for the treatment of patients with lymphoid malignancies, where this pro-survival protein is commonly over-expressed [1]. Among the Bcl-2 inhibitors developed so far, venetoclax appears to be the most promising compound [1]. The development of the pan-Bcl-2 inhibitor obatoclax has been ceased due to low response rates and neurologic and psychiatric side effects [1,2,32], and the use of navitoclax, which not only inhibits Bcl-2 but also Bcl-x_L_ and Bcl-w, is limited due to acute, dose-dependent thrombocytopenia [1,2,32]. Although mono-therapy is also effective, the combination of venetoclax with other antineoplastic agents is considered more effective, and on-going clinical trials currently address this question [2]. Combination with other than cytostatic drugs is also likely to treat symptoms or comorbidities in cancer patients [33]. However, there are so far no data published at all concerning possible pharmacokinetic drug–drug interactions of venetoclax. We therefore underwent *in vitro* investigations to determine whether venetoclax influences the expression or activity of drug-metabolizing enzymes and drug transporters determining pharmacokinetics and whether it retains its efficacy in MDR cell lines.

Our data demonstrate that venetoclax inhibits the drug transporters P-gp, BCRP, OATP1B1, and OATP1B3 and the drug metabolizing enzymes CYP2C19 and CYP3A4 with a moderate potency compared to known strong inhibitors (Table 1). Thus, the question of whether these effects will also play a role in the clinical situation arises. Therapeutic plasma concentrations of venetoclax reach about 5 µM [3,34,35], a similar range to concentrations inhibiting drug transporters and CYP *in vitro*. Moreover, in the intestine, even higher concentrations are reached (about 1 mM after a dose of 250 mg according the formula published by the Food and Drug Administration (FDA) [36]), making an inhibition of transporters and CYPs expressed there (like P-gp, CYP3A4, and CYP2C19 [37]) very likely. Following the suggestion of the FDA, drugs for which [I]_1_/IC_50_ > 1 or [I]_2_/IC_50_ > 10 (with [I]_1_ = mean steady-state *C*_max_ for total drug; [I]_2_ = dose of inhibitor/250 mL) should be evaluated *in vivo* to determine whether a clinically relevant P-gp inhibition occurs. Thus, even the moderate inhibition of P-gp by venetoclax might be relevant and should be tested *in vivo*.

Whereas drug–drug interactions provoked by inhibition of drug metabolizing enzymes and drug transporters can lead to potentiation of the effects of co-administered drugs, induction of these proteins might result in reduced drug effects. We therefore also investigated whether venetoclax can induce important phase I and phase II enzymes as well as drug transporters modulating pharmacokinetics. While mRNA expressions of *CYP3A4*, *ABCC2*, and *ABCG2* were unchanged, venetoclax increased mRNA expression of *CYP1A1*, *CYP1A2* (also verified at the protein level), *UGT1A3*, and *UGT1A9* at higher concentrations (10 µM), which are also in the range of therapeutic plasma concentrations [34,35]. Because all these genes are transcriptionally regulated by AhR [38,39,40], we also evaluated whether venetoclax activates AhR, but the reporter gene assay clearly demonstrated that ventoclax is not an activator of AhR. Thus, the underlying mechanism for the mRNA increase is most likely post-transcriptional.

An interesting finding of potential relevance is the observed pronounced decrease of the mRNA and protein expression of P-gp by venetoclax. Suppression of P-gp might be an advantage in combination therapy because many cytostatic drugs are P-gp substrates and lose their efficacy when P-gp is over-expressed (MDR) [41]. Suppression of P-gp expression and activity (as demonstrated in the P-gp inhibition assay) might therefore preserve the efficacy of P-gp substrates in combination with venetoclax. It might also be advantageous because our data indicate that venetoclax is a P-gp substrate, as shown by the fact that the P-gp over-expressing cell line L-MDR1 was more resistant towards venetoclax than its parental counterpart, and this resistance could be abolished with the specific P-gp inhibitor LY335979. This points to a reduced efficacy of venetoclax itself in the presence of P-gp-mediated MDR. However, this might be of no importance in the clinical situation because venetoclax suppresses P-gp expression and activity, thus possibly abolishing the MDR status.

Our data demonstrate a slight reduction of *SLCO1B1* mRNA expression by venetoclax. However, this reduction was only observed at 5 µM and not at lower or higher concentrations, thus questioning the relevance of this effect.

Limitations: (1) We have only analyzed a selection of drug metabolizing enzymes and drug transporters. Other proteins might also contribute to drug–drug interactions with venetoclax. (2) We have investigated the influence of venetoclax on mRNA and protein expression only in one cell system. The extent of mRNA/protein changes might be different in other cell lines. (3) Only for P-gp and CYP1A2 mRNA changes were verified at the protein level. However, variations in mRNA levels commonly translate into changes of the corresponding protein or altered function as shown in this study for P-gp and CYP1A2.

## 5. Conclusions

In conclusion, our study scrutinized the interaction profile of venetoclax *in vitro*. Our data indicate that venetoclax might act as a perpetrator drug (inhibitor or inducer) with other drugs being substrates of CYP1A, CYP2B6, CYP2C19, CYP3A4, UGT1A3, UGT1A9, and P-gp. Whether venetoclax can modulate P-gp-mediated MDR in addition to Bcl-2 inhibition requires further *in vivo* studies.

## Figures and Tables

**Table 1:** IC_50_ values for transporter and CYP inhibition by venetoclax.

Protein Inhibited	Venetoclax [µM]	Control Compound	[µM]
P-gp	30.0 ± 3.7	Verapamil	2.9 ± 0.8 [29]
BCRP	19.6 ± 7.3	FTC	0.7 ± 0.3 [30]
OATP1B1	47.8 ± 10.1	Rifampicin	2.4 ± 0.9 [12]
OATP1B3	26.0 ± 9.6	Rifampicin	2.1 ± 1.0 [12]
CYP1A2	no inhibition		
CYP2B6	activation		
CYP2C19	14.21 ± 1.0	Omeprazole	0.8 ± 0.2 [31]
CYP2D6	no inhibition		
CYP3A4	7.2 ± 3.2	Ketoconazole	0.035 ± 0.015 [31]
